# Research progress on the mechanisms and animal models of Ménière’s disease

**DOI:** 10.1016/j.gendis.2025.102022

**Published:** 2026-01-02

**Authors:** Siyuan Liu, Yanshi Li, Yuting Zhang, Yuxiao Zheng, Chen Jin, Lin Chen, Guohua Hu, Wenqi Zuo

**Affiliations:** a The First Affiliated Hospital of Chongqing Medical University, Chongqing 400016, China; b Department of Audiology and Speech-Language Pathology, Chongqing Medical University, Chongqing 401331, China

**Keywords:** Animal models, Biomarker, Ménière’s disease, Mechanism studies, Pathogenesis

## Abstract

Ménière’s disease is a prevalent chronic condition that triggers a range of vestibular and auditory symptoms. Despite significant advances in mechanisms such as genetic susceptibility, immunity, and allergy, a clear understanding of its core pathophysiology has not been established, and a consistent theoretical framework is still lacking. In this review, we discuss the advantages and disadvantages of recent animal models of Ménière’s disease, advances in mechanistic studies on Ménière’s disease, and current treatment approaches. This discussion aims to provide a reference for future research on the mechanisms of Ménière’s disease.

## Introduction

According to the World Health Organization, at least 700 million people will suffer from varying degrees of hearing loss by 2050.^1^ Many factors contribute to hearing loss, including noise exposure, viral infections, and tumors 2, 3, 4. However, the causes of many inner ear diseases remain enigmatic. For example, the primary pathological feature of Ménière’s disease (MD) is endolymphatic hydrops (EH). Episodic vertigo, fluctuating hearing loss, tinnitus, and aural fullness comprise the core symptoms first documented by Prosper Ménière.^5^ The prevalence of this disease varies depending on the geographical and demographic background of patients, ranging from approximately 3 to 513 cases per 100,000 population.^6^ Moreover, the prevalence of MD increases with age and is significantly higher among women than in men.^7^ In recent years, research into the pathogenesis of MD has implicated diverse factors, including immune dysregulation, viral infections, acute inflammation, inner ear ischemia, genetics, and Ca^2+^ overload 8, 9, 10. A growing paradigm now suggests that rather than acting independently, these disparate triggers may converge upon a common pathological hub: the endolymphatic sac (ES). Thus, while the clinical pathology of MD manifests in the vestibular and cochlear systems, the aforementioned factors inducing ES may serve as the starting point triggering the entire disease cascade.^11^

## Animal models of MD

In recent years, various scholars have focused on EH to establish animal models of MD, aiming to further explore its pathogenic factors and treatment options (Table 1). Currently, modeling methods can be classified based on the speed of induction into acute and chronic models, or categorized as non-surgical models induced by systemic administration of vasopressin, aldosterone, or postauricular injection of lipopolysaccharide,^12^^,^^13^ and surgical models involving artificial endolymph injection into the cochlea.^14^ Neither a single surgical lesion nor an isolated biological agent injection can fully replicate the pathogenesis of MD. In the future, the combination of surgical disruption and biological agent injections may help elucidate the pathogenic mechanisms mediated by different etiological factors in sporadic MD. Additionally, some scholars have attempted to construct EH models using gene knockout techniques, such as conditional knock-out of SOX9/SOX10 genes^15^ to establish MD models. Beyond rodent models, researchers have successfully developed Drosophila models by targeting homologs of human MD-risk genes.^16^ Furthermore, the zebrafish has emerged as another promising model organism, owing to the high homology of its genome to genes associated with familial MD, suggesting its significant potential for research on hereditary MD.^17^ Unfortunately, these modeling methods often lead to permanent hearing loss and vestibular dysfunction, thereby failing to fully replicate the clinical manifestations of MD, such as fluctuating hearing loss and recurrent vertigo.

**Table 1:** Different modeling methods for Ménière’s disease.

Inventors	Animal models		Assessment			Limitations
	Animals	Methods	Hearing symptoms	Vestibular symptoms	Pathology	
Feldman et al^88^	Guinea pig	Cholera toxin injection into the scala media induced time-dependent changes in the endolymphatic potential, which were assessed at 45, 75, and 120 min post-injection	Absence of symptoms	Absence of symptoms	Hydrops	Only EH was noted
Salt et al^89^	Guinea pig	Slow injection of 0.5–2 μL sodium hyaluronate gel into the cochlear apex was performed while continuously recording changes in the endocochlear potential, summating potential, and transducer operating point	Changes in physiological parameters	Absence of symptoms	Hydrops	The modeling method was complex, and no direct manifestation of hearing impairment
Katagiri et al^90^	Adult CBA/J or ICR mice	Subcutaneous injection of vasopressin was administered for 5 days to 8 weeks	Partial loss of associated cells	Impaired balance reflexes	Hydrops	No direct manifestation of hearing impairment
Zhang et al^13^	C57BL/6 mice	Bilateral post-auricular sustained infusion of lipopolysaccharide was carried out for 3 days, with functional validation performed on day 5	Elevated hearing thresholds	Impaired balance reflexes	Hydrops	No fluctuating hearing loss was detected
Egami et al^91^	Guinea pig	Functional evaluation was conducted at 1 or 4 weeks after endolymphatic sac electrocauterization, with desmopressin administered subcutaneously 1 h before testing to trigger vestibular symptoms	Absence of symptoms	Impaired balance reflexes	Hydrops	The modeling method was complex, and no hearing-related symptoms were present
Dunnebieret al^92^	Guinea pig	During the third week following endolymphatic sac ablation by different approaches, the animals received a daily intraperitoneal injection of aldosterone at 100 pg/100 g for five consecutive days, followed by histological evaluation	Changes in the morphology of the associated cells	Absence of symptoms	Hydrops	The modeling method was complex, and no direct manifestation of hearing impairment
Watanabe et al^93^	Guinea pig	Guinea pigs were primed by an intraperitoneal injection of 500 μg keyhole-limpet hemocyanin (KLH) in complete Freund’s adjuvant. Fourteen days later, their endolymphatic sacs were infused with 100 μg of KLH in 5 μl of PBS via an intradural glass micropipette. Histological assessment was performed 24 h after the infusion	Associated cells show iNOS immunoreactivity	Absence of symptoms	Hydrops	The modeling method was complex, and no direct manifestation of hearing impairment
Valenzuela et al^94^	Guinea pig	The endolymphatic sac was destroyed, and the endolymphatic duct was injured using a Fine pick, and the modeling outcome was evaluated on postoperative day 30	While the cochlear compound action potential thresholds at frequencies of 8 kHz and above remained within normal limits in the majority of modeled Guinea pigs, the thresholds of the auditory nerve overlapped waveform showed an increase	Absence of symptoms	Hydrops	This modeling method is advantageous due to its short duration, minimal confounding effects, and high success rate (86%). However, it does not include an assessment of vestibular function
Ye et al^95^	C57BL/6J mice	EH was induced in C57BL/6J mice by daily intraperitoneal injection of vasopressin at a dose of 50 μg/100 g for 2 or 4 weeks.	Significant hearing loss, measured by auditory brainstem response, emerged at 4 weeks, whereas distortion product otoacoustic emissions revealed earlier cochlear dysfunction, beginning with low-frequency loss at 2 weeks and progressing to widespread damage by 4 weeks	Absence of symptoms	Hydrops	Despite the advantages of low cost and technical simplicity, this model is limited by the availability of only a single evaluation method
Requena et al^16^	Drosophila	A Drosophila model of Ménière’s disease was established through mutation of the Dystrobrevin gene. Phenotypic validation involved a longitudinal assessment of auditory and balance functions at multiple time points (2, 10, 20, and 30 days post-eclosion).	Reduced auditory gain and decreased the number of sensitive mechanically conductive pathways	The mutant flies exhibited a significant defect in climbing ability	Sertoli cell functional deficiency is associated with the disruption of the hemolymph-neuron barrier and neuronal stress	This model allows for longitudinal assessment of auditory and vestibular function. However, its relevance to human Ménière’s disease pathophysiology requires further validation

Current validation of these models primarily relies on functional assays like auditory brainstem response and vestibular evoked myogenic potentials. While existing models, such as the lipopolysaccharide-induced one, were not designed to test the ES hypothesis, they can be repurposed to investigate immune-mediated ES dysfunction. Therefore, future model development should incorporate the evaluation of ES pathology as a core validation metric. This, however, first requires establishing objective and standardized histopathological criteria.

## Biomolecular and differentially expressed genes related to MD

**Figure 1:**
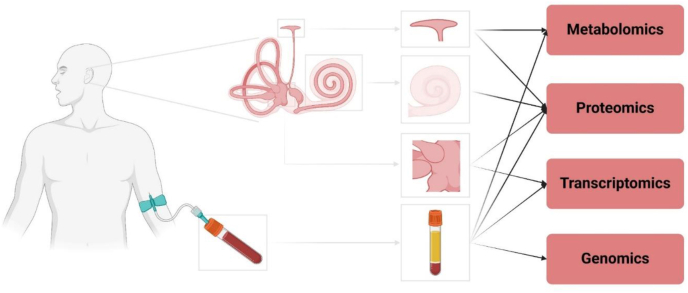
Summary of current sequencing methods for patients with Ménière’s disease.

Due to the presence of the inner ear barrier, the vestibular window, and the round window, it is challenging to access the complex microenvironment of the inner ear using conventional techniques and to study biomarkers. This has resulted in limited predictive validity and reliability of diagnostic and detection methods for MD, thereby restricting the efficacy of clinical treatment. However, the advent of multi-omics technologies is now overcoming this barrier (Fig. 1), providing unprecedented molecular evidence that powerfully supports the central role of the ES as a pathological hub (Table 2). The following sections synthesize findings from metabolomics, proteomics, transcriptomics, and genomics, demonstrating how diverse molecular perturbations ultimately converge on ES dysfunction.

**Table 2:** Integration of multi-omics evidence with key pathophysiological pathways in Ménière’s disease.

Candidate pathways	Key molecules/biological processes	Omics evidence	Primary affected inner ear structures
Disruption of ion homeostasis	Ca^2+^ homeostasis; K^+^ cycle; AQP2; KCNE gene family	Metabolomics^18^: Altered levels of citrate and EDTA in endolymphatic sac fluid.	Stria vascularis/endolymphatic sac
		Genomics^44^: Allelic variants of KCNE3 and AQP2	
Abnormal immune signaling	Complement system activation; pro-inflammatory cytokines; MHC class II molecules	Proteomics^23^: Complement activation in endolymphatic sac tissue; elevated levels of IL-1β, IL-6, and TNF-α in plasma.	Endolymphatic sac/vestibular end organs
		Transcriptomics^29^: Up-regulation of immune-related genes in peripheral blood mononuclear cells; loss of MHC class II tolerance.	
Extracellular matrix remodeling	Hyaluronic acid metabolism; collagen and structural proteins	Metabolomics^18^: Elevated hyaluronic acid in endolymphatic sac fluid.	Endolymphatic sac
		Single-cell sequencing^37^: Ribosome-rich cells are enriched in extracellular matrix pathways.	
Synaptic dysfunction	Neurotransmitter release; neuroimmune interaction	Proteomics^25^: Alterations in synapse-associated proteins within the vestibular end organs.	Vestibular hair cells/Spiral Ganglion neurons
		Transcriptomics^30^: Expression of neuropathy-associated genes in vestibular tissue.	

### Metabolomics

Metabolomic studies offer a direct readout of the biochemical activity within the inner ear, and their findings are highly consistent with the proposed role of the ES in maintaining inner ear fluid homeostasis. Huang et al performed ES decompression on 10 patients with intractable MD and aspirated 2 μL of diluted ES fluid. Subsequent liquid chromatography-mass spectrometry analysis demonstrated that, compared with the control group, the levels of hyaluronic acid, 4-hydroxynonenal, and 2,3-diaminopropanoate were significantly elevated in the ES fluid of patients with MD. However, the levels of citrate, ethylenediaminetetraacetic acid, and d-glucuronic acid were markedly reduced. These findings suggest that MD is closely associated with oxidative damage, inflammatory lesions, and disturbances in endolymphatic Ca^2+^ homeostasis.^18^ This directly implicates the ES as an important site of failed ion regulation. This aligns with the earlier finding by Ishiyama et al, who reported elevated levels of iNOS and nitrotyrosine in vestibular tissue specimens from patients with MD, supporting the hypothesis that oxidative stress plays a key role in the disease.^19^ While oxidative stress is not pathognomonic for MD, its detection in the ES underscores this structure’s vulnerability and central position in the disease cascade, potentially acting as a common downstream effector for various upstream insults.^20^^,^^21^

### Proteomics

In early studies, some scholars screened a group of patients with MD based on the 1995 AAO-HNS guidelines and compared them with healthy controls (matched by sex and age). The results showed that patients with MD exhibited elevated expression of complement factor H/B, fibrinogen alpha, and gamma chains, and significantly reduced levels of β-2 glycoprotein-1, vitamin D-binding protein, and apolipoprotein-1 in their plasma.^22^ In a separate study,^23^ Chiarella et al used an enzyme-linked immunosorbent assay to demonstrate significantly elevated concentrations of pro-inflammatory cytokines, including interleukin-1β (IL-1β), interleukin-1 receptor antagonist, interleukin-6 (IL-6), and tumor necrosis factor-α (TNF-α), in the supernatant of approximately 21% of patients. Additionally, some researchers collected inner ear perilymph and identified unique protein expressions in patients with MD, such as short-chain dehydrogenase/reductase family 9C member 7.^24^ However, it is noteworthy that these early studies, whether analyzing plasma or perilymph, only indirectly reflect systemic variations resulting from inner ear pathology in patients with MD, which may not fully or promptly capture molecular changes within the inner ear tissues. The endolymphatic fluid is enclosed within the membranous labyrinth, where direct sampling of cochlear and vestibular structures is ethically impermissible due to the high risk of perilymph–endolymph contamination. Additionally, most patients retain residual hearing and may be candidates for future cochlear implantation, rendering intraoperative cochlear duct resection for pathological analysis clinically unjustifiable. Notably, recent advances in high-sensitivity mass spectrometry have enabled the analysis of partial vestibular tissues and ultralow-volume endolymphatic fluid samples from the ES.

In a recent study by Zhang et al,^25^ researchers performed direct proteomic analysis of tissue samples obtained from affected membranous labyrinth structures, specifically the vestibular end organs and ES, in patients with MD. The results suggested that the affected vestibular end organs were highly associated with changes in synaptic-related neurotransmitters, while the ES proteomic data are consistent with the findings of Xiong’s team,^26^ suggesting that local activation of the complement system and the programmed cell death it triggers may constitute a key component of ES immunoregulation. This finding positions the ES as an active site of immune dysregulation, where complement-mediated processes may represent a key pathway through which diverse triggers (*e.g.*, infection, genetics) could initiate the disease cascade.

### RNA sequencing

Current research on the transcriptomics of MD primarily focuses on peripheral blood mononuclear cells of patients. For instance, research groups led by Lopez-Escamez et al and Sun et al ^23^^,^^27^^,^^28^ conducted RNA sequencing on peripheral blood mononuclear cells from patients with MD. Their results revealed elevated expression levels of multiple genes, including IGHG1, KRT72, IGLV3-21, TMEM176B, and TMEM176A. Additionally, the RNA-sequencing analysis of patients with MD by Choi et al^29^ suggested that the collapse of MHC class II-mediated immune tolerance may contribute to the development of MD. These findings collectively indicate that MD is associated with immune pathways. Furthermore, a study collected vestibular organs (utricle, saccule, and vestibular cristae) from patients with late-onset MD and performed RNA sequencing. Through Gene Ontology and Kyoto Encyclopedia of Genes and Genomes pathway enrichment analyses of differentially expressed genes, it was found that, compared with patients with acoustic neuromas and facial neuromas, many differentially expressed genes (*e.g.*, MAG, MPZ, and CLDN19) were associated with cell adhesion, autoimmune diseases, and neuropathy.^30^ Notably, the transcriptomic analysis of MD vestibular tissues did not align with the aforementioned proteomic findings. We speculate three possible reasons for this discrepancy: i) Differences in disease subtypes: Transcriptomic studies in the above literature have focused on late-onset MD, whereas proteomic studies have mainly dealt with sporadic MD. ii) Post-transcriptional regulatory mechanisms may disrupt the correlation between transcript and protein abundance. A key example is miRNA-mediated silencing,^31^ which is predicted to target genes such as glutathione peroxidase, and this could substantially contribute to the observed discrepancies.^32^^,^^33^ iii) Significant inter-sample heterogeneity remains even among patients of the same clinical subtype. First, we acknowledge the presence of elevated pro-inflammatory factors in some patients with MD. As some reports suggest, individuals with autoimmune deficiencies, such as systemic lupus erythematosus, rheumatoid arthritis, and psoriasis, are more likely to develop MD.^9^ However, a multicenter retrospective study^34^ indicates that approximately 53.4% of patients have sporadic MD, and elevated pro-inflammatory factors during the quiescent phase of sporadic MD are rare. Moreover, the detection of elevated pro-inflammatory factors during active phases does not necessarily support the notion that autoinflammation causes MD, as acute infections or vertigo episodes can also lead to increased pro-inflammatory factors.^35^ Therefore, inflammation is more likely a key driver and amplifier of the disease process rather than a universal initiating event.

Radiological imaging of the temporal bone has shown that the ES may undergo different pathological changes in various clinical subtypes of MD, such as hypoplasia or degeneration, with degenerative changes in the ES epithelium being a primary feature.^36^ It is well known that the ES epithelium consists of mitochondria-rich cells interspersed among ribosome-rich cells, with approximately 70% of the epithelial cells being ribosome-rich and 30% being mitochondria-rich.^37^ However, the heterogeneity of ES epithelial cells has limited recent research into the mechanisms of MD-related hydrops. The functional differences between epithelial cells due to the abundance of specific organelles are yet to be unraveled. In 2023, Szeto et al^15^ performed single-cell sequencing on ES cells from an MD animal model. Their results suggest that mitochondria-rich epithelial cells are associated with membrane transport proteins and ion channels involved in maintaining inner ear fluid homeostasis, while ribosome-rich epithelial cells are enriched in pathways related to the extracellular matrix. This functional segregation provides a precise cellular mechanism for the ES hub hypothesis: genetic or molecular insults could disrupt ion transport mediated by mitochondria-rich cells (leading to endolymphatic imbalance) and/or extracellular matrix maintenance mediated by ribosome-rich cells (impairing structural integrity), collectively culminating in EH. Although the modeling method used in this study has certain limitations, the sequencing results significantly align with the differential expression profiles of ES from patients with MD directly measured by Zhang’s team.^25^ Thus, it is reasonable to conclude that this approach demonstrates the potential to effectively characterize selected aspects of MD behavior. Although mice and guinea pigs have been widely used in ontological research, significant species differences between humans and animals remain. Aside from studies in animal models, no published research to date has reported single-cell sequencing of the ES in patients with MD.

### Genomics

The advent of whole-exome sequencing and whole-genome sequencing has provided reliable tools for studying the genetic factors of MD. Based on the current research, the MD-related genes are primarily familial MD (FMD)-related genes and sporadic MD (SMD)-related genes. The known FMD-related genes are summarized in Table 3. FMD is defined as cases where at least one relative (first- or second-degree) of the patient shows clinical symptoms that meet all diagnostic criteria for MD. Most FMD cases exhibit autosomal dominant inheritance, while a few families show autosomal recessive inheritance or mitochondrial inheritance.^38^ On the contrary, SMD is defined as cases that meet all diagnostic criteria for MD without migraines or autoimmune inflammation.

**Table 3:** Possible candidate genes for familial Ménière’s disease.

Author and year	Study design	Population	Gene or region	Inheritance pattern	Role of candidate gene or region	Single-nucleotide variants
Requena et al, 2015^96^	Familial	Spanish	FAM136A DTNA	Autosomal dominant inheritance	DTNA can encode a membrane protein that interacts with the cytoskeleton, involved in the formation and stability of synapses. FAM136A’s physiological function has not yet been determined	2:70527974G > A 18:32462094G > T
Martín-Sierra et al, 2016^97^	Familial	Spanish	PRKCB	Autosomal dominant inheritance	PRKCB may play a role in supporting the function of cells in the organ of Corti, particularly in maintaining the endolymphatic potassium (K^+^) cycle	16:23999898G > T
Martín-Sierra et al, 2017^98^	Familial	Spanish	DPT SEMA3D	Autosomal dominant inheritance	DPT, a protein that interacts with cell surface integrins and proteoglycans, can encode dermatopontin. SEMA3D encodes Semaphorin-3D, an axonal guidance protein involved in the regulation of cytoskeletal dynamics and cell adhesion	1:168665849G > A 7:84042128G > A
Skarp et al, 2019^99^	Familial	Finn	HMX2 TMEM55B	Autosomal recessive inheritance	HMX2 is involved in the development of the inner ear and vestibular structures in mice and zebrafish. TMEM55B participates in the regulation of cell membrane structure and cytoskeleton formation	10:124909634T > A 14:20927370G > A
Mehrjoo et al, 2020^100^	Familial	Iranian	LSAMP	Autosomal recessive inheritance	LSAMP is a neuronal surface glycoprotein, although its role in the innervation of the organ of Corti and vestibular organs remains to be elucidated	3:115561402T > C
Roman-Naranjo et al, 2020^101^	Familial	Spanish	OTOG	Autosomal recessive inheritance	OTOG encodes otogelin, a protein expressed in the sensory epithelium of the inner ear. This protein is involved in the pathophysiological processes underlying hearing and vestibular function, and its dysfunction is associated with deafness and balance disorders	11:17594747C > A 11:17621218C > T 11:17663747G > A
Roman-Naranjo et al, 2021^102^	Familial	Spanish Switzerland	MYO7A	Autosomal recessive/dominant inheritance	MYO7A encodes a motor protein that is essential for the organization and maintenance of stereocilia in auditory and vestibular hair cells	11:76841683G > A 11:76870496G > A 11:76885923G > A 11:76890920G > A 11:76891450C > T 11:76910646G > A 11:76922875G > A 11:76925719G > A 11:76925733G > A
Roman-Naranjo et al, 2022^103^	Familial	Spanish	TECTA	Autosomal dominant inheritance	TECTA encodes α-tectorin, a key extracellular matrix protein that mediates stereociliary deflection and regulates the gating of mechanotransduction channels	11:121158016T > C 11:121152980G > C 11:121165368C > T

Epidemiological studies indicate that FMD accounts for approximately 10% of MD cases, and OTOG, MYO7A, and TECTA are the most common mutated genes.^39^ A summary of recently reported candidate genes for FMD is presented in Table 2, including their functional roles and single-nucleotide variants. Some studies suggest that hearing loss in patients with FMD appears to be associated with abnormalities in stereocilia and tectorial membrane function. However, the candidate genes currently linked to FMD lack large-scale validation and consistent detection rates. For example, most reported FMD cases involve single-nucleotide variants. Nearly all the associated genes have been identified exclusively in single families, without validation through cross-family case–control studies. However, only the same single-nucleotide variant in the OTOG gene has been reported in multiple independent families.^40^ Additionally, the candidate genes described in Table 2 are almost exclusively derived from whole-exome sequencing. Future research should not overlook introns and non-coding regions, as structural variations in these areas may also play a critical role in FMD.^41^ Finally, the candidate genes for FMD require further validation through animal models and functional cellular studies. In summary, these candidate genes are primarily expressed in neural or inner ear tissues, suggesting that FMD may affect the normal function of inner ear hair cells through abnormal neural discharge or neurotransmitter release.

Compared with the screening of candidate genes for FMD, the SMD research tends to favor genome-wide association studies, which can detect single-nucleotide polymorphisms across the entire genome. Based on the histopathological characteristics of EH and the hypothesis of endolymphatic fluid circulation disorders, current scholars believe that candidate genes for SMD are related to ion channels, immune responses, and aquaporins. Discussions on the phenomenon of EH primarily focus on two aspects: either it may result from the overactivation of tissues with fluid secretion functions, such as the stria vascularis^42^; or, it may be due to functional abnormalities in areas proven to have fluid absorption capabilities, such as the ES and tissues containing dark cells.^43^ Three main hypotheses have been proposed: i) Endolymphatic fluid passively enters and exits the inner ear membranous labyrinth through aquaporins. ii) Endolymphatic fluid is passively transported through active transport/facilitated diffusion via ion channels, as seen in Pendred syndrome. iii) Endolymphatic fluid moves through intercellular spaces in the inner ear’s membranous labyrinth. Early studies identified KCNE3 allele mutations in patients with chronic tinnitus.^44^ It is known that the KCNE gene family plays a significant role in cardiac diseases.^45^ Additionally, it has been reported that patients with long QT syndrome carry KCNE1 gene mutations, which are strongly associated with congenital deafness.^46^ Additionally, some scholars screened the KCNE genes in patients with MD and found that 492 KCNE3 A/C single-nucleotide polymorphisms showed statistically significant differences between the FMD group and the control group,^44^ suggesting that KCNE genes may act as susceptibility genes for SMD. In 2020, Gallego-Martinez et al^47^ conducted targeted gene sequencing on 860 SMD patients and identified missense variants in the NTN4 gene (rs537136429, rs34114770, and rs199956955), implying that axon guidance signaling may induce SMD. Furthermore, many researchers have also performed targeted sequencing on immune-related genes^47^^,^^48^ and aquaporins, discovering that some allele mutations may be associated with MD. In 2019, Gallego-Martinez et al^49^ also identified mutations in hearing-related genes, such as SLC26A4, CLDN14, and GJB2, in Spanish patients with MD. Previous studies have shown up-regulated expression of aquaporin-2 (AQP2) on the basolateral side of ES epithelial cells in patients with MD,^50^ along with elevated vasopressin levels in plasma. Based on these findings, it is hypothesized that ES epithelial cells may regulate endolymphatic fluid homeostasis through the AVP–V2R–AQP2 pathway, similar to the kidneys.^51^ However, elevated plasma AVP levels appear to be associated with bilateral MD, whereas most patients exhibit unilateral symptoms. Recent studies have shown no increase in plasma AVP levels in patients with MD, which may be related to less stringent inclusion criteria in earlier studies. This contradiction suggests that certain factors may independently regulate AQP2 translocation in patients with MD.

In summary, genomic studies, particularly in SMD, are identifying genetic susceptibilities that converge on biological processes—ion transport, immune regulation, and fluid dynamics—that are central to the postulated functions of the ES. This provides the heritable blueprint that predisposes this structure to become the disease’s common bottleneck.

### Non-omics research on biomolecules in MD

A well-established correlation between MD and allergic reactions demonstrates that atopic patients show increased susceptibility to MD.^52^ Repeated allergen exposure may contribute to the fluctuating hearing loss characteristic of early-stage MD. As a specific biomarker of type I hypersensitivity, IgE has been implicated in MD pathogenesis. Additionally, clinical studies demonstrate that anti-IgE therapy can alleviate MD symptoms.^53^ Recent studies have identified IgE deposits in vestibular tissues, and their density was found to positively correlate with both the degree of EH and hearing loss thresholds. This observation suggests that the up-regulation of IgE and CD23 expression in the inner ear may be mediated by IL-4, IL-5, and IL-13.^54^ Moreover, it has been hypothesized that IgE-mediated activation of mast cells and basophils triggers IL-4 release, promoting Th2 cell differentiation and subsequent M2 polarization of macrophages.^55^ Further clinical observations reveal that patients with acute low-frequency sensorineural hearing loss commonly display elevated IgE levels, with 31% subsequently developing MD.^56^ Although IgE holds potential as a diagnostic biomarker, comprehensive multicenter studies remain necessary to confirm its spatiotemporal expression profile within the inner ear microenvironment.

**Figure 2:**
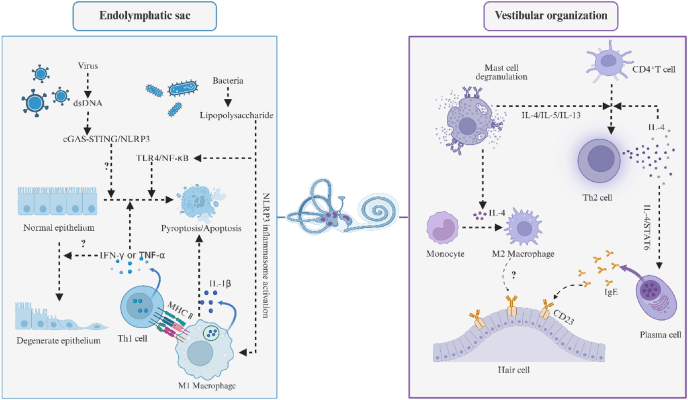
Hypothetical schematic diagram of the immune mechanism in Ménière’s disease.

As illustrated in Figure 2, the ES is regarded as the principal immunologic tissue^57^ that shields the inner ear from pathogenic invasion and has been central to MD research. Initial investigations revealed the presence of macrophages, T cells, and plasma cells within the sac, lending credence to the immunological basis of MD.^58^ Subsequent findings demonstrated elevated concentrations of TNF-α and IFN-γ^59^ in ES cavity fluid, likely secreted by T cells. This confirms the hypothesis that immune mechanisms modulate MD progression. A meta-analysis^60^ established an approximately threefold association between human cytomegalovirus infection and MD relative to controls, with antiviral therapy proving efficacious in a subset of patients, potentially through the recognition of viral dsDNA by innate immune pathways such as cGAS-STING,^61^ though definitive viral infection markers remain elusive. Most recently, Wang et al^62^ identified correlations among MD, inner ear hypoxia, and middle ear anaerobic bacteria, proposing a pathogenic mechanism whereby anaerobic bacteria may infiltrate the ES via the round or oval window to mediate MD development, suggesting these bacteria could serve as prospective diagnostic markers for MD.

## Central system disorder in MD

When unilateral peripheral vestibular damage occurs, it can trigger a series of vestibular symptoms, such as vertigo, postural instability, and vomiting. These symptoms often gradually resolve on their own within days to weeks; this phenomenon is known as “vestibular compensation”. Vestibular compensation is a highly complex process that typically involves the coordinated or competitive actions of multiple central brain regions, including the vestibular nuclear complex, vestibulocerebellum, and cerebral cortex.^63^ The recurrent episodes of vertigo in MD may be related to abnormal vestibular excitability. Gentamicin, an aminoglycoside antibiotic, exhibits selective toxicity toward vestibular hair cells in the inner ear. Clinically, intratympanic injection of gentamicin is used to destroy vestibular hair cells, particularly type I vestibular hair cells,^64^ thereby reducing abnormal excitability of the vestibular nerve on the affected side and allowing the central nervous system to recalibrate balance function through its plasticity mechanisms. This approach helps alleviate vertigo symptoms in patients with MD. A retrospective study found that the hippocampal volume in patients with MD was significantly reduced compared with healthy controls, and the degree of hippocampal volume loss correlated with the severity of hearing and vestibular dysfunction.^65^ Recently, it has been reported that the extent of hippocampal atrophy in patients with MD is significantly associated with EH.^66^ Additionally, some studies suggest that patients with late-onset MD are more prone to developing dementia,^67^ and they are more likely to exhibit cognitive deficits compared with those with benign paroxysmal positional vertigo.^68^ This may occur because damage to the peripheral vestibular system in patients with MD leads to dysregulation of the glutamatergic system within the hippocampus, which in turn mediates hippocampal atrophy. Based on this evidence, we propose a mechanistic model in which peripheral vestibular damage in MD triggers dysregulation of the hippocampal glutamatergic system,^69^ and the subsequent glutamate excitotoxicity drives hippocampal atrophy.^70^

## Potential mechanisms of treatment for MD

### Acute phase

During the acute phase of MD, the primary focus is on controlling vertigo while preserving inner ear function to a maximum extent. Clinically, vestibular suppressants such as antihistamines and anticholinergics are commonly used. These drugs may work by blocking vestibular nerve activity and receptors in the vestibular nuclear complex.^71^ However, they do not directly address vertigo by targeting and modulating vestibular hair cells.^72^ Additionally, intratympanic injections of glucocorticoids have shown good efficacy in controlling vertigo.^73^ Recent studies suggest that dexamethasone may inhibit the formation of inflammasomes in inner ear macrophages through glucocorticoid-induced kinase 1, thereby down-regulating the expression of IL-1β, TNF-α, and other inflammatory factors in the membranous labyrinth of patients with MD.^35^ However, these studies do not explain how pro-inflammatory factors contribute to the development of EH.

### Intermittent phase

The therapeutic approach for MD focuses on reducing or preventing vertigo. Currently, oral medications include betahistine and diuretics. Primarily, betahistine binds to central histamine receptors, influencing the discharge rates of bilateral vestibular nuclear complexes, and ultimately achieving a rebalancing of vestibular function on both sides. Previous studies suggest that betahistine may promote neuronal depolarization by activating hyperpolarization-activated cyclic nucleotide-gated channels in the lateral vestibular nucleus^74^ and by activating H1 receptors on GABAergic neurons in the medial vestibular nucleus on the affected side, thereby increasing inhibition of GABAergic neurons in the contralateral medial vestibular nucleus.^75^ On the contrary, diuretics are generally believed to be related to aquaporin-2 in the basal cells of the stria vascularis and the ES. They may reduce endolymphatic volume by promoting endolymph drainage or decreasing its production in the stria vascularis.^76^

Surgical interventions can be employed to alleviate vertigo in patients with intractable MD. Common methods include endolymphatic sac decompression (ESD), labyrinthectomy, vestibular nerve section, and semicircular canal occlusion.^77^^,^^78^ However, the mechanisms underlying these procedures are not yet fully understood. In 2015, Saliba et al^79^ introduced endolymphatic duct blockage (EDB), a novel surgical approach for treating MD. Several retrospective studies have demonstrated that this method effectively relieves vertigo in patients with no significant side effects.^80^^,^^81^ This raises a new question, as the principles of ESD and EDB are contradictory. ESD is based on the assumption that the endolymphatic sac absorbs endolymphatic fluid, while EDB operates on the premise that the ES may secrete endolymphatic fluid and that the endolymphatic duct might have fluid absorption capabilities. This discrepancy highlights the superficial nature of our current understanding of ES function and suggests that MD may be a heterogeneous disease driven by multiple pathogenic mechanisms. A summary of common interventions for MD, including their proposed mechanisms, risks, patient selection criteria, levels of evidence, and expected outcomes, is provided in Table 4.

**Table 4:** Interventions for Ménière’s disease: Mechanisms and clinical application.

Interventions	Potential Mechanisms of Action	Risks and Disadvantages	Patient Selection	Level	Expected effect
Betahistine	Acts on the vestibular nuclei to rebalance intervestibular function	It may be ineffective and can delay other necessary treatments	Applicable to patients at all disease stages, particularly during the interictal period	B^104^	Therapeutic efficacy is uncertain
Diuretics	Systemic dehydration, and potentially by modulating the AQP2 water channels in the stria vascularis and endolymphatic sac, to reduce endolymphatic volume	Electrolyte disturbances, hypotension, fatigue	Patients in the interictal period who are willing and able to tolerate the side effects and undergo regular monitoring	B^104^	May reduce attack frequency, but the benefit is not confirmed by high-quality randomized controlled trials
Intratympanic steroids	Diffuses into the inner ear via the round window membrane to suppress local inflammation (*e.g.*, by reducing IL-1β and TNF-α) and stabilize the inner ear environment	It requires multiple injections and carries the risks of tympanic membrane perforation	Patients with unilateral disease, significant hearing fluctuations, refractory to conservative management, and who desire hearing preservation	C^104^	Improved vertigo control
Intratympanic gentamicin	Selectively ablates secretory vestibular dark cells and hair cells, thereby reducing pathological signaling input	It entails a definite risk of hearing loss	Patients with refractory unilateral Ménière’s disease that has failed all other treatments	B^104^	High vertigo control rate
ESD/EDB	ESD: Postulates a dysfunction in endolymph absorption by the sac; surgery aims to improve absorption via decompression. EDB: Postulates that the sac/duct may secrete harmful substances or exhibit abnormal absorption; the procedure aims to interrupt this process by obstruction	General surgical risks, with unpredictable postoperative efficacy	Refractory patients who have failed conservative management, desire hearing preservation, and are unwilling to undergo destructive procedures	ESD: C^104^/EDB: Unavailable	ESD: Two-year control rate 80%–90%/EDB: The complete control rate of postoperative vertigo is approximately 42%.^105^
Vestibular nerve Section (VNS)	Severs the vestibular nerve to permanently block the transmission of abnormal signals from the affected ear to the brain	VNS carries a risk of complications and complicates potential future vestibular implantation	Patients with refractory unilateral Ménière’s disease and disabling vertigo who are willing to accept the risks of intracranial surgery	C^104^	Vertigo control rate: 78%–90%
Semicircular canal plugging	Plugs the semicircular canal to prevent endolymph flow from impacting the ampullary crest, thereby eliminating the aberrant motion signals	Persistent balance disorders and vestibular hypofunction	Single or total semicircular canal plugging for Ménière’s disease	Unavailable	The successful control rate of vertigo was 100% in the average 23-month postoperative follow-up period^106^

Note: All evidence in Table 4 pertaining to levels and expected effects, except that for EDB and semicircular canal plugging, originates from the 2020 AAO-HNS Clinical Practice Guideline: Ménière’s Disease. ESD, endolymphatic sac decompression; EDB, endolymphatic duct blockage.

### Dietary and nutritional interventions in MD

An unhealthy diet and inadequate nutritional intake have been reported as one of the triggering factors for MD.^82^ Consumption of high-sodium foods can amplify MD symptoms, whereas a low-salt diet appears to be an effective management strategy for patients with MD.^83^ This may be associated with the expression of Na^+^/Cl^–^ cotransporter,^84^ Na^+^/K^+^ ATPase,^84^ and ENaC^85^ in ES epithelial cells. Additionally, a case–control study found^86^ a correlation between caffeine intake and MD. Since caffeine contains methylxanthines, excessive caffeine exposure may induce vasoconstriction and ischemia in the inner ear, ultimately leading to cochlear damage. Although research on the relationship between diet and MD remains limited,^87^ maintaining a low-salt diet and restricting caffeine and alcohol intake are recommended.

## Perspectives and conclusions

In conclusion, we propose a unifying hypothesis for MD pathogenesis: despite the clinical heterogeneity driven by diverse triggers (*e.g.*, immune, inflammatory), the initial insult consistently occurs at the ES, and it is the resultant ES pathology that initiates the disease cascade. Validating this model requires mapping the molecular time-series of inner ear cell types during EH formation using single-cell and spatial genomics. Thus, the mainstream of future MD research should focus on combining histologic validation with functional experiments on ES epithelial cells and vestibular hair cells to define the precise mechanisms operating within this framework.

## CRediT authorship contribution statement

**Siyuan Liu:** Writing – original draft, Visualization, Data curation, Conceptualization. **Yanshi Li:** Writing – review & editing, Supervision, Investigation. **Yuting Zhang:** Resources, Investigation. **Yuxiao Zheng:** Visualization, Resources, Investigation. **Chen Jin:** Resources, Investigation. **Lin Chen:** Writing – review & editing, Visualization. **Guohua Hu:** Supervision, Investigation, Funding acquisition. **Wenqi Zuo:** Writing – review & editing, Supervision, Project administration, Funding acquisition.

## Funding

This work was supported by the 10.13039/501100005230Natural Science Foundation of Chongqing, China (No. CSTB2024NSCQ-MSX1014).

## Conflict of interests

The authors declared no conflict of interests. Figures were created with biorender.com.
